# Optimizing therapy for relapsed/refractory classic Hodgkin lymphoma in the era of PD‐1 blockade

**DOI:** 10.1002/hem3.70110

**Published:** 2025-05-12

**Authors:** Thomas M. Kuczmarski, Ryan C. Lynch

**Affiliations:** ^1^ Division of Hematology/Oncology University of Washington, Fred Hutchinson Cancer Center Seattle Washington USA

Most patients with relapsed/refractory (R/R) classic Hodgkin lymphoma (CHL) are cured with primary therapy, but 10%–30% of patients may relapse.^1^, ^2^, ^3^ While second‐line salvage chemotherapy followed by high‐dose chemotherapy and autologous stem cell transplant (ASCT) may cure most R/R patients, the optimal salvage treatment regimen remains undefined. With the advent of targeted therapies, novel agents such as the CD30 antibody‐drug conjugate brentuximab vedotin (BV) as well as PD‐1 inhibitors such as nivolumab and pembrolizumab have been incorporated into salvage regimens.^4^, ^5^, ^6^, ^7^ Acknowledging the limitations of cross‐trial comparison of phase 2 trials, the outcomes with these regimens appear superior to using chemotherapy alone.^8^, ^9^

In this issue of *HemaSphere*, Mei et al. present the results of their phase 2 trial of combination nivolumab plus ifosfamide, carboplatin, and etoposide (NICE) in patients with high‐risk R/R Hodgkin lymphoma.^10^ The study was performed in light of an initial PET‐adapted approach, in which patients received either nivolumab monotherapy or combination NICE (for patients with residual PET‐positive disease after nivolumab lead‐in) followed by ASCT.^5^ Given that only 9 of 43 enrolled patients received NICE in the PET‐adapted approach, this study was performed to further evaluate the safety and efficacy of NICE before ASCT. In total, 35 patients were enrolled in the study. All patients received one dose of nivolumab monotherapy followed by two cycles of NICE, and seven patients received a third cycle of NICE. Notably, this cohort treated with NICE was restricted to high‐risk individuals based on several pre‐determined criteria, including relapse within 1 year of completion of first‐line therapy or primary refractory disease.

In this study, NICE followed by ASCT demonstrated promising efficacy with a 2‐year progression‐free survival (PFS) and overall survival (OS) of 88% and 100%, respectively. These efficacy data are comparable to other studies in which a combination of PD‐1 inhibitor and chemotherapy was used in the R/R setting.^4^, ^11^ Given that the cohort was restricted to patients with high‐risk diseases, it makes the efficacy results even more compelling.

While the study demonstrated promising efficacy data, it also determined the regimen to be safe. In fact, toxicities associated with NICE followed by ASCT were similar to if not better than prior studies involving combination chemoimmunotherapy.^4^, ^6^, ^11^ Anemia (69%) and nausea (69%) were the most common toxicities attributed to NICE, while transaminitis (23%), rash (20%), and pruritus (14%) were the most common immune‐related adverse events (all grade 1, except for two cases of pruritus that were grade 2). Rates of neutropenia and thrombocytopenia were relatively low at 31% with the majority of cases grade 2 or lower.

One important toxicity‐related clinical question that this study helps answer is whether prior exposure to PD‐1 inhibitor is associated with increased rates of engraftment syndrome during ASCT. For context, one study of pembrolizumab‐GVD followed by ASCT yielded rates of engraftment syndrome of 68%, and another study of 42 patients treated with pembrolizumab‐ICE followed by ASCT had one fatal case of engraftment syndrome.^4^, ^6^ Encouragingly, rates of engraftment syndrome in this study were modest (14%), and more in line with the prior PET‐adapted NICE study (12%) and a retrospective analysis of patients treated with PD‐1 inhibitor before ASCT (18%).^5^, ^12^

While this study highlights the efficacy and safety of combination PD1 inhibitor and chemotherapy in the R/R setting, it also raises important clinical questions. The S1826 study demonstrated a 2‐year PFS of 92% for patients treated with nivolumab‐AVD in untreated advanced‐stage CHL, meaning the use of frontline PD‐1 inhibitor‐based regimens will become more common.^3^ When patients develop R/R disease after prior frontline exposure to PD‐1 inhibitors, what is the utility of subsequent PD‐1‐based regimens in the R/R setting? This is one area in which further research is warranted, as there are limited data to guide clinical practice in this situation. Patients with clear refractory disease (e.g., relapse <3 months) to frontline PD1‐inhibitor‐based therapy should receive a salvage regimen containing BV (Figure 1). Recent long‐term follow‐up of a dose‐dense combination of BV and ICE showed a 5‐year PFS of 77%, albeit in patients with no previous exposure to any novel agent.^7^, ^13^

**FIGURE 1:**
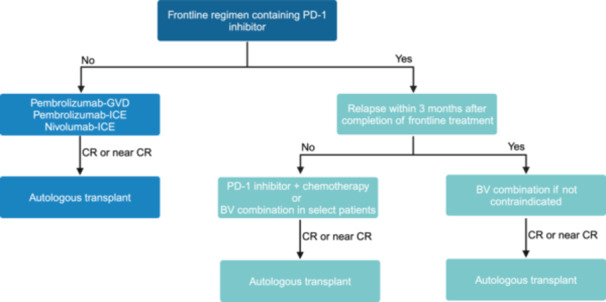
Management of transplant‐eligible relapsed/refractory classic Hodgkin lymphoma in the era of PD‐1 blockade.

Where the uncertainty lies is in the optimal salvage regimen for patients who relapse later (e.g., >3 months after frontline treatment). Should these patients receive another PD‐1‐inihbitor‐based regimen? Patients who discontinue pembrolizumab or nivolumab after achieving a CR can be re‐challenged again upon progression and achieve a response.^14^, ^15^ On top of that, can sensitivity to PD‐1 blockade be reinvigorated through the use of immunostimulatory drugs? In a heavily pre‐treated cohort of 13 patients with R/R CHL, a combination of gemcitabine and pembrolizumab exhibited an objective response of 85%, with five patients achieving a complete response.^16^ Ten out of 11 patients who had experienced progressive disease on PD‐1 blockade immediately before switching to gemcitabine‐pembrolizumab demonstrated a response, suggesting that gemcitabine may augment response to PD‐1 blockade through immunostimulatory mechanisms and that immunostimulatory medications could play an important role in the R/R setting. How this applies to patients with later relapse after frontline PD1‐inhibitor therapy is unknown at this time, and real‐world data in the coming years will have to answer this question. In the meantime, one can still consider BV‐based salvage in this setting given the high cure rates seen in long‐term follow‐up.^13^ But similarly, real‐world data will help define the efficacy of this regimen in those who relapse after PD‐1‐inhibitor‐based frontline therapy.

While the majority of patients in the study by Mei et al. proceeded to ASCT,^10^ it is also important to question whether ASCT in this setting is even necessary. One recent study demonstrated a 2‐year PFS of 51% for 40 patients who were treated with pembrolizumab maintenance alone after achieving a complete remission after four cycles of pembrolizumab‐GVD.^17^ This study highlights that combination chemoimmunotherapy followed by PD‐1 blockade monotherapy may be sufficient treatment for a subset of patients—likely those without stage IV disease—and that a substantial portion of patients may not need ASCT at all. When PD‐1 blockade is used in conjunction with chemotherapy, it is also unclear if there is an optimal chemotherapy backbone—GVD or ICE—and future randomized comparison trials with integrated correlation with ctDNA for measurable residual disease assessment should explore this further.^18^, ^19^, ^20^

In patients with R/R disease without prior PD‐1 blockade exposure, it is clear that integrating PD‐1 blockade into the salvage regimen is critical. The study by Mei et al. provides further evidence for this approach.^10^ Future studies should focus on optimizing salvage regimens when PD‐1 blockade was previously used as well as defining the population that may benefit from ASCT in the R/R setting.

## CONFLICT OF INTEREST STATEMENT

T. M. K.: None. R. C. L.: Research Funding: TG Therapeutics, Incyte, Bayer, Cyteir, Genentech, Pfizer, Rapt, Merck, Janssen, and Allogene; Consultancy/honoraria: SeaGen, AbbVie, Janssen, Merck, and ADC Therapeutics.

## FUNDING

This research received no funding.

## AUTHOR CONTRIBUTIONS

**Thomas M. Kuczmarski**: Conceptualization; writing—original draft; writing—review and editing. **Ryan C. Lynch**: Conceptualization; writing—original draft; writing—review and editing.

## Data Availability

Data sharing is not applicable to this article.
